# Prevalence and risk factors of shoulder stiffness after rotator cuff repair: a meta-analysis

**DOI:** 10.1186/s12891-026-09856-0

**Published:** 2026-05-05

**Authors:** Xin-Lei Tang, Yi-Lin Wang, Zhong-You Zhang, Xuan Cheng, Si-Yuan Gao, Sheng Ding

**Affiliations:** 1 Guanghua Hospital Affiliated to Shanghai University of Traditional Chinese Medicine, NO.1508, Yan’an West Road, Shanghai, Shanghai 200052 China; 2 Shanghai University of Traditional Chinese Medicine, No.1200, Cailun Road, Shanghai, Shanghai 201203 China

**Keywords:** Prevalence, Risk factors, Rotator cuff, Postoperative shoulder stiffness, Meta-analysis

## Abstract

**Background:**

This meta-analysis aims to determine the prevalence of postoperative shoulder stiffness (PSS) after rotator cuff repair surgery and to explore its potential risk factors.

**Methods:**

Search the PubMed, Embase, Web of Science, and Cochrane Library databases from their inception to July 1, 2025, for all studies reporting the prevalence of PSS after rotator cuff repair surgery and its associated risk factors. The Newcastle–Ottawa Scale (NOS) was used to assess the quality of the included studies. Meta-analysis was performed using Stata 15.0 software.

**Results:**

Eight cohort studies involving 21,033 patients were included. The pooled prevalence of postoperative shoulder stiffness (PSS) after rotator cuff repair was 17% (95% CI: 9%–24%), with substantial heterogeneity (I^2^ = 98.2%). Subgroup analysis by continent showed variation in reported prevalence estimates across regions, although these findings should be interpreted cautiously because some subgroups were based on a limited number of studies. Additional subgroup analyses according to sample size, publication year, and mean age were conducted to explore heterogeneity. In the sample size subgroup analysis, the pooled prevalence was 14% (95% CI: 2%–25%) in studies with a sample size ≥ 300 and 18% (95% CI: 15%–21%) in studies with a sample size < 300, with lower heterogeneity in the latter subgroup. By contrast, subgroup analyses by publication year and mean age did not materially reduce heterogeneity. In pooled analyses of adjusted effect estimates, age < 50 years (OR = 1.09, 95% CI: 1.03–1.15), female sex (OR = 1.66, 95% CI: 1.03–2.68), and diabetes mellitus (OR = 2.73, 95% CI: 1.75–4.26) were associated with PSS; however, the effect size for age < 50 years was small.

**Conclusion:**

PSS after rotator cuff repair is not uncommon, but the pooled prevalence should be interpreted cautiously because of substantial heterogeneity and inconsistent diagnostic definitions across studies. Female sex and diabetes mellitus may be associated with PSS, whereas the clinical relevance of the association with age < 50 years appears limited. Further high-quality prospective studies are required.

**Supplementary Information:**

The online version contains supplementary material available at 10.1186/s12891-026-09856-0.

## Background

The rotator cuff is a crucial structure for maintaining shoulder joint stability and enabling complex shoulder movements [1]. It consists of four muscles—the supraspinatus, infraspinatus, teres minor, and subscapularis—and their corresponding tendons [2]. With the aging of the population and changes in lifestyle, the incidence of rotator cuff injuries has been rising annually, making it one of the most common shoulder conditions in clinical practice [3]. According to reports, the incidence of rotator cuff tears in the elderly population can exceed 25%, and among middle-aged and young adults engaged in repetitive shoulder activities or physically demanding work, the incidence is also showing a rising trend [4]. If left untreated, rotator cuff tears often lead to shoulder pain, functional impairment, and muscle atrophy, significantly impacting patients’ quality of life [5]. Therefore, rotator cuff repair surgery, as an effective treatment modality, has been widely adopted in clinical practice [5].

With the advancement of arthroscopic techniques, the safety and efficacy of rotator cuff repair surgery have continued to improve. Its minimally invasive nature and rapid recovery time have made it the current standard procedure [6]. However, despite the increasing maturity of surgical techniques, postoperative complications remain a significant concern, with postoperative shoulder stiffness (PSS) being one of the most common and troublesome complications [7, 8]. PSS is one of the most common and clinically relevant complications after rotator cuff repair. In addition to limiting shoulder motion, PSS may impair activities of daily living, delay return to work and recreational activities, prolong rehabilitation, and reduce patient satisfaction with surgery. For surgeons, this complication is particularly important because it can complicate postoperative recovery despite technically successful repair and may necessitate additional treatment, including extended physiotherapy, corticosteroid injection, manipulation under anesthesia, or arthroscopic capsular release. PSS may therefore increase healthcare utilization and impose a substantial functional and socio-economic burden on both patients and healthcare systems. A clearer understanding of its prevalence and associated factors is thus important for surgical counseling, risk stratification, and postoperative management [8]. Its clinical manifestations include a marked restriction in the range of motion of the shoulder (especially abduction, flexion, and rotation), with or without pain. In severe cases, it can lead to long-term functional impairment and may even require reoperation [9]. The pathogenesis of PSS has not yet been fully elucidated and may involve postoperative inflammatory responses, scar formation, joint capsule contracture, and soft tissue adhesions [10]. Some patients experience transient stiffness in the early postoperative period, which can gradually improve with rehabilitation therapy; while others develop persistent joint stiffness, resulting in prolonged rehabilitation periods or even incomplete recovery [11, 12]. Relevant literature indicates that the occurrence of PSS not only affects patient satisfaction and functional recovery but also significantly increases the medical burden. Therefore, investigating the incidence of PSS and its risk factors is of great significance for optimizing preoperative assessment, developing individualized postoperative rehabilitation strategies, and improving patient outcomes [13, 14].

Currently, several studies have examined the incidence of shoulder stiffness following rotator cuff repair surgery and its associated risk factors; however, the results exhibit significant heterogeneity and controversy [15, 16]. On one hand, the definition criteria for PSS vary across different studies. For example, some studies use reduced active range of motion as the criterion, while others employ passive range of motion or imaging-based criteria, leading to poor comparability of results. On the other hand, differences in the demographic characteristics of study participants, surgical techniques (single-row repair vs. double-row repair), and postoperative rehabilitation protocols also influence the reported incidence of PSS. Additionally, regarding potential risk factors for PSS, previous studies have proposed various possibilities, including gender, age, surgical technique, preoperative activity restrictions, diabetes, thyroid disease, and postoperative fixation duration [17, 18]. However, there is currently a lack of systematic, quantitative, and integrated evidence to support these findings.

PSS following rotator cuff repair is a common, complex, and clinically significant issue that impacts prognosis. However, the true prevalence of this condition and its primary risk factors remain controversial, with no consensus reached to date. Therefore, it is necessary to conduct a systematic review and meta-analysis to integrate relevant research data and obtain more representative and reliable conclusions. This study aims to address this research gap, clarify the epidemiological status of PSS, identify high-risk populations, and provide evidence-based guidance for clinical practice, with the goal of improving postoperative recovery quality and reducing the incidence of related complications.

## Methods

The program was constructed in accordance with the Preferred Reporting Items for Systematic Reviews and Meta-Analyses (PRISMA) guidelines [19]. This study was pre-registered with the International prospective register of systematic reviews (PROSPERO) under the registration number: CRD420251061067.

### Inclusion and exclusion criteria

#### Inclusion criteria

The study subjects were adult patients who underwent rotator cuff repair surgery; The study reported the incidence of PSS or related risk factors; The study type was a cohort study; Sufficient statistical data were provided for meta-analysis, such as prevalence, baseline differences between the PSS group and the non-PSS group, or odds ratios (OR) and related 95% confidence intervals (CI).

#### Exclusion criteria

Study subjects were non-adult patients (children or adolescents); Study subjects were patients who underwent other types of shoulder surgery, or those with conditions such as simple shoulder arthritis or subacromial bursitis that did not involve rotator cuff repair; The incidence of shoulder stiffness was not reported, or there was a lack of data on relevant risk factors; The study was a case report or a small-sample study (< 5 participants), and could not provide the data required for statistical analysis; The study was not an original study, such as a review, expert opinion, or conference abstract.

### Literature retrieval

A comprehensive literature search was conducted in multiple electronic databases, including PubMed, Embase, Web of Science, and the Cochrane Library, from database inception to July 1, 2025. The search strategy combined keywords related to “risk factors,” “rotator cuff injuries,” and “stiffness,” using Boolean operators (AND, OR) to ensure comprehensive retrieval. The detailed search strategy for each database is provided in Supplementary Material Table S1.

### Study collection and extraction

Two reviewers independently screened all retrieved studies. After removal of duplicates, titles and abstracts were assessed to exclude irrelevant studies. The full texts of potentially eligible studies were then reviewed for final inclusion. Any disagreements were resolved through discussion with a third reviewer.

Data extraction was performed independently by two reviewers using a predefined data extraction form. Extracted variables included first author, publication year, country, study design, sample size, number of patients with PSS, sex distribution (male/female), mean age, and regression model. For risk factor analyses, only multivariable-adjusted ORs and their 95% confidence intervals were extracted and pooled in order to reduce the influence of confounding. A third reviewer verified the extracted data for consistency. When necessary, attempts were made to contact the original authors to obtain missing data. Studies with insufficient data were excluded from the analysis.

### Assessment of risk of bias

The methodological quality of the included studies was assessed independently by two reviewers using the Newcastle–Ottawa Scale (NOS). The NOS evaluates studies across three domains: selection, comparability, and exposure (or outcome), with a maximum score of 9 points. Studies scoring 7–9 were considered high quality, 5–6 moderate quality, and 0–4 low quality. No studies were excluded solely based on quality assessment; instead, study quality was considered in the interpretation of the results [20].

### Statistical analysis

All statistical analyses were performed using Stata version 15.0 (StataCorp, College Station, TX, USA). For prevalence outcomes, a single-proportion meta-analysis was conducted. To stabilize the variance of proportions, a transformation method (Freeman–Tukey) was applied prior to pooling, and pooled estimates were back-transformed to the original scale for presentation. Given the anticipated clinical and methodological heterogeneity across studies, a random-effects model was used for all meta-analyses. Statistical heterogeneity was assessed using Cochran’s Q test and the I^2^ statistic, with I^2^ > 50% indicating substantial heterogeneity.

Subgroup analyses were performed to explore potential sources of heterogeneity. However, given the limited number of studies and variability in reporting, these analyses were considered exploratory. Sensitivity analysis was conducted using a leave-one-out approach to evaluate the robustness of the pooled estimates.

Publication bias was assessed using funnel plots and Egger’s test. A *P* value < 0.05 was considered indicative of potential publication bias. Where appropriate, the trim-and-fill method was applied to explore the possible impact of missing studies.

## Results

### Literature retrieval results

**Fig. 1:**
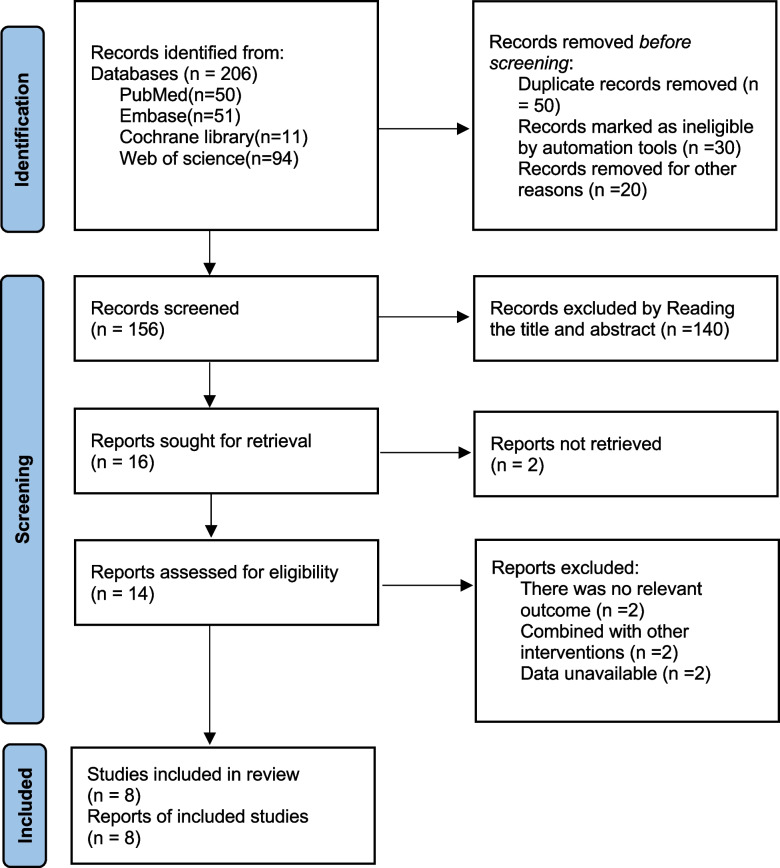
Literature search flowchart. PRISMA 2020 flow diagram for new systematic reviews which included searches of databases and registers only. *Consider, if feasible to do so, reporting the number of records identified from each database or register searched (rather than the total number across all databases/registers). **If automation tools were used, indicate how many records were excluded by a human and how many were excluded by automation tools. *From:* Page MJ, McKenzie JE, Bossuyt PM, Boutron I, Hoffmann TC, Mulrow CD, et al. The PRISMA 2020 statement: an updated guideline for reporting systematic reviews. BMJ 2021;372:n71. 10.1136/bmj.n71

As shown in Fig. 1, a total of 206 articles were retrieved from PubMed (*n* = 50), Embase (*n* = 51), Cochrane Library (*n* = 11), and Web of Science (*n* = 94). After removing 50 duplicate articles, 140 articles were excluded based on title and abstract review, and 6 articles were excluded after full-text review. Ultimately, 8 articles [21–28] were included in the analysis.

### Basic characteristics of the included literature

This study included a total of 8 cohort studies involving 21,033 patients with an average age of 58–60 years. The studies were conducted in the United States, South Korea, Germany, Australia, Iran, China, and Colombia. The specific characteristics of the studies are shown in Table 1.

**Table 1:** Characteristics of the included studies

Study	Year	Study design	Country	Sample size	NO of Postoperative Stiffness	Gender(M/F)	Mean age	Regression model	Surgical technique	Tear characteristics reported	Rehabilitation protocol	Timing of stiffness assessment	Definition of postoperative shoulder stiffness
Burrus	2019	cohort study	USA	19,229	232	NR	NR	multivariate logistic regression	Arthroscopic	NR/mixed tear types	NR	Up to 9 months postoperatively	Stiffness requiring MUA and/or LOA
Cho	2022	cohort study	Korea	274	39	104/160	60.1	multivariate logistic regression	Arthroscopic	Tear size, synovitis, fatty infiltration	Passive ROM at 2–4 weeks; active ROM at 6 weeks	3 months	Passive FF < 120° or ER < 30°
Chung	2013	cohort study	Korea	288	50	128/160	59.53	Multivariate Logistic Regression	Arthroscopic	Tear size, fatty infiltration, retear	Immediate passive ROM; strengthening at 9–12 weeks	3, 6 months, and final follow-up	Passive FF < 120°, ER < 30°, or IR < L3
Cucchi	2022	cohort study	Germany	385	19	146/239	59.7	Multivariate Logistic Regression	Arthroscopic	Tear type (partial vs complete), SCOI classification	Passive ROM early; structured rehab protocol	≥ 90 days	Passive ER < 10° (side) or < 30° (90° abduction), or FF < 100°
Dannaway	2024	cohort study	Australia	250	41	192/58	58	Multivariate Logistic Regression	Arthroscopic	Tear size, HbA1c, tear type	Immobilization 6 weeks; gradual ROM thereafter	3 and 6 months	Passive ER at side < 30°
Guity	2021	cohort study	Iran	335	121	128/207	68.5	Multivariate Logistic Regression	Arthroscopic	Tear size, trauma vs degenerative	Passive ROM after 2 weeks; strengthening after 2 months	3 months	Total passive ROM deficit (25–70° moderate; > 70° severe)
Li	2024	cohort study	China	155	39	54/101	58.1	Multivariate Logistic Regression	Arthroscopic	Tear size, comorbidities	NR/standard rehab	Within 6 months	FF < 120° or ER < 30°, lasting > 12 weeks
Salas	2024	cohort study	Colombia	117	23	48/69	59	Multivariate Logistic Regression	Arthroscopic	Tear size, retear, comorbidities	Early passive motion; structured rehab	3 and 6 months	≤ 15th percentile of FF and/or ER

*NR* Not reported

### Risk of Bias results

This study used the NOS score, and the results (Table 2) showed that five articles [21, 24–26, 28] scored 9 points, two articles [22, 23] scored 8 points, and one article [27] scored 7 points, indicating that the overall quality of the included studies was high.

**Table 2:** Quality assessment of included studies using the Newcastle–Ottawa Scale (NOS)

Cohort study									
Study	Representativeness of the exposed group	Selection of non-exposed groups	Determination of exposure factors	Identification of outcome indicators not yet to be observed at study entry	Comparability of exposed and unexposed groups considered in design and statistical analysis	design and statistical analysis	Adequacy of the study’s evaluation of the outcome	Adequacy of follow-up in exposed and unexposed groups	Total scores
Burrus 2019 [21]	*	*	*	*	**	*	*	*	9
Cho 2022 [22]	*	*	*	/	**	*	*	*	8
Chung 2013 [23]	*	*	*	/	**	*	*	*	8
Cucchi 2022 [24]	*	*	*	*	**	*	*	*	9
Dannaway 2024 [25]	*	*	*	*	**	*	*	*	9
Guity 2021 [26]	*	*	*	*	**	*	*	*	9
Li 2024 [27]	*	*	*	/	**	/	*	*	7
Salas 2024 [28]	*	*	*	*	**	*	*	*	9

### Meta-analysis results

All pooled risk factor estimates were derived from adjusted effect estimates reported in the original studies.

### Prevalence rate

**Fig. 2:**
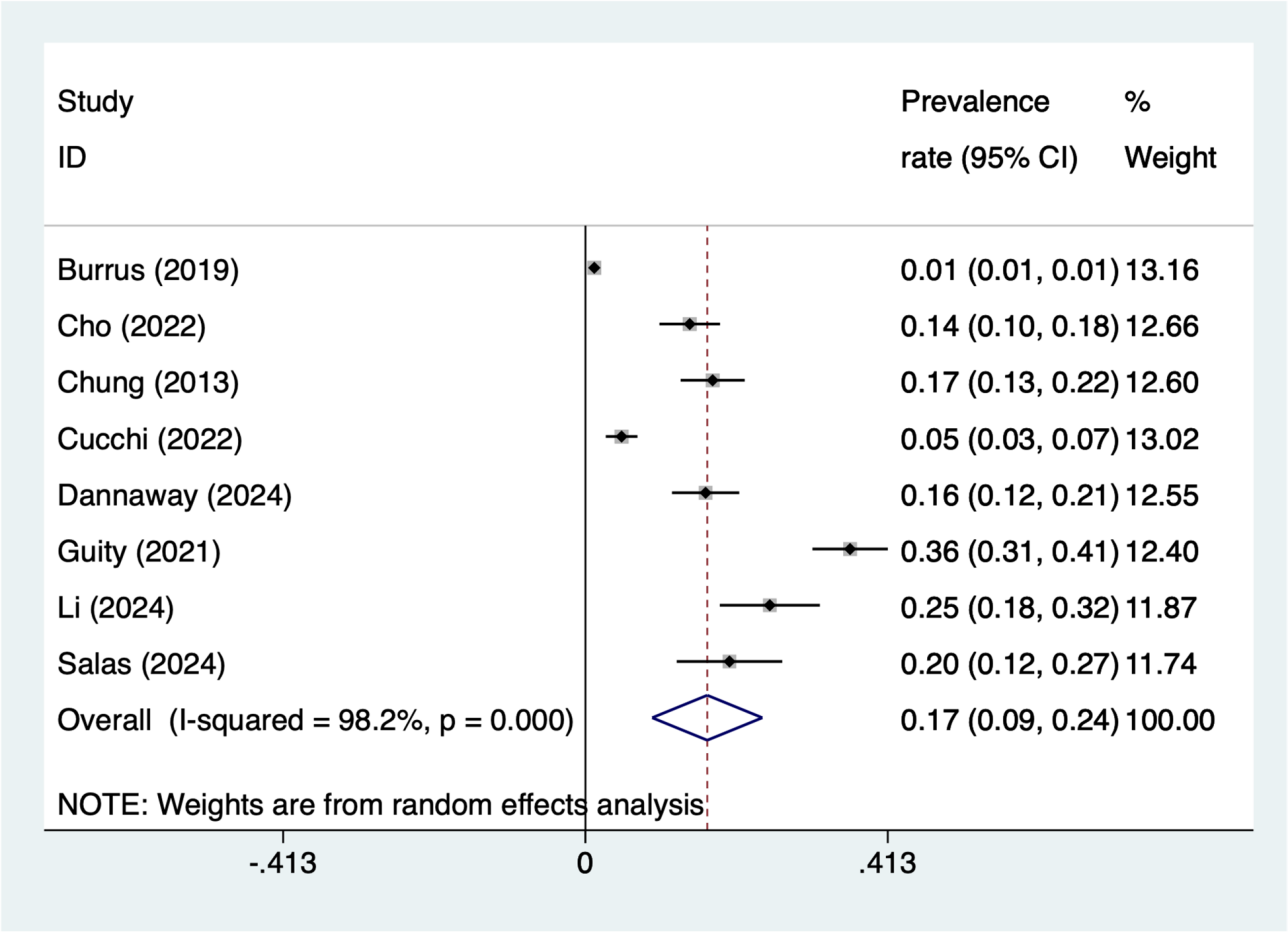
Forest plot of the pooled prevalence of postoperative shoulder stiffness (PSS) after rotator cuff repair. Each square represents the prevalence estimate from an individual study, with the size of the square reflecting the relative study weight. Horizontal lines indicate 95% confidence intervals (CIs). The diamond represents the pooled prevalence estimate and its 95% CI. Prevalence estimates to the right indicate a higher proportion of PSS. Abbreviations: PSS, postoperative shoulder stiffness; CI, confidence interval

Eight studies reported the prevalence of PSS. Considerable heterogeneity was observed across studies (I^2^ = 98.2%, *P* < 0.001). Using a random-effects model, the pooled prevalence of PSS after rotator cuff repair was 17% (95% CI: 9%–24%) (Fig. 2). Sensitivity analysis was performed by sequentially omitting individual studies, and the results suggested that the pooled estimate was not driven by any single study (Supplementary Material Figure S1).

Subgroup analysis (Table 3) by continent showed prevalence by continent showed prevalence estimates of 10% in the Americas, 23% in Asia, 5% in Europe, and 16% in Oceania. To further explore potential sources of heterogeneity, additional subgroup analyses were conducted according to sample size, publication year, and mean age. In the sample size subgroup analysis, the pooled prevalence was 14% (95% CI: 2%–25%; I^2^ = 98.9%) among studies with a sample size ≥ 300 and 18% (95% CI: 15%–21%; I^2^ = 48.4%) among those with a sample size < 300. In the publication year subgroup analysis, the pooled prevalence was 9% (95% CI: −7% to 25%; I^2^ = 98.1%) in studies published before 2020 and 19% (95% CI: 10%–29%; I^2^ = 96.6%) in studies published in or after 2020. In the mean age subgroup analysis, the pooled prevalence was 17% (95% CI: −2% to 36%; I^2^ = 99.1%) among studies with a mean age ≥ 60 years and 16% (95% CI: 8%–24%; I^2^ = 93.9%) among those with a mean age < 60 years. Nevertheless, substantial between-study variability remained, likely reflecting differences in study populations, definitions of postoperative shoulder stiffness, surgical techniques, rehabilitation protocols, and timing of assessment. These findings suggest that the pooled prevalence should be interpreted cautiously and may not reflect the prevalence in any specific clinical setting.

**Table 3:** Subgroup analysis results

Subgroup variable	Subgroup	No. of studies	Pooled prevalence (95% CI)	I^2^ (%)	P for heterogeneity
Continent	Americas	2	10% (6%–28%)	82.1	< 0.001
	Asia	4	23% (13%–33%)	75.2	< 0.001
	Europe	1	5% (3%–7%)	NR	< 0.001
	Oceania	1	16% (12%–21%)	NR	< 0.001
Sample size	≥ 300	3	14% (2%–25%)	98.9	< 0.001
	< 300	5	18% (15%–21%)	48.4	0.101
Publication year	< 2020	2	9% (−7% to 25%)	98.1	< 0.001
	≥ 2020	6	19% (10%–29%)	96.6	< 0.001
Mean age	≥ 60 years	3	17% (−2% to 36%)	99.1	< 0.001
	< 60 years	5	16% (8%–24%)	93.9	< 0.001

### Age < 50

**Fig. 3:**
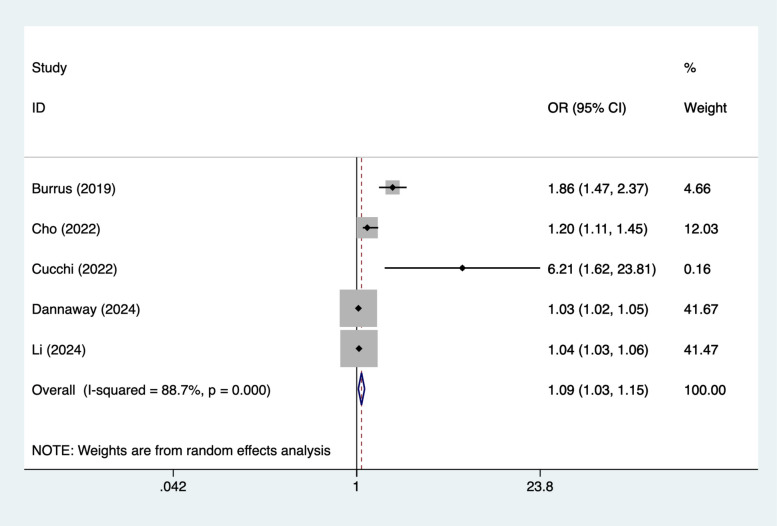
Forest plot of the association between age < 50 years and postoperative shoulder stiffness (PSS) after rotator cuff repair. Each square represents the odds ratio (OR) from an individual study, with square size proportional to study weight. Horizontal lines indicate 95% confidence intervals (CIs). The diamond represents the pooled OR and its 95% CI. An OR > 1 suggests that age < 50 years was associated with higher odds of PSS, whereas an OR < 1 suggests lower odds

Five studies reported data for age < 50 years. Substantial heterogeneity was observed (I^2^ = 88.7%, *P* < 0.001). Using a random-effects model, age < 50 years was associated with PSS after rotator cuff repair (OR = 1.09, 95% CI: 1.03–1.15) (Fig. 3). Sensitivity analysis by sequential omission suggested that the pooled estimate was not driven by any single study (Supplementary Material Figure S2). However, given the modest effect size and substantial heterogeneity, this finding should be interpreted cautiously.

### Female sex

**Fig. 4:**
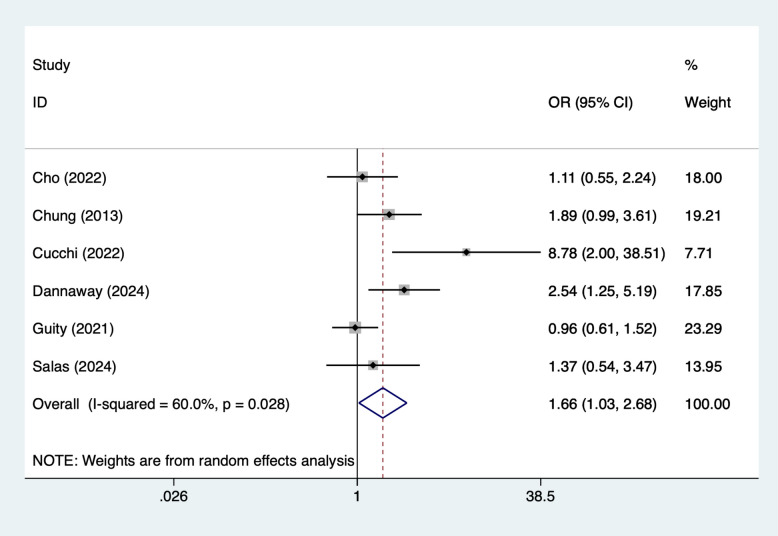
Forest plot of the association between female sex and postoperative shoulder stiffness (PSS) after rotator cuff repair. Each square represents the odds ratio (OR) from an individual study, with square size proportional to study weight. Horizontal lines indicate 95% confidence intervals (CIs). The diamond represents the pooled OR and its 95% CI. An OR > 1 suggests that female sex was associated with higher odds of PSS

Six articles mentioned female sex. A heterogeneity test (I^2^ = 60%, *P* = 0.028). A random-effects model was used for analysis. The results (Fig. 4) suggest that female sex was associated with PSS after rotator cuff repair [OR = 1.66, 95% CI (1.03, 2.68)]. A sensitivity analysis was conducted by sequentially excluding studies. The results (Supplementary Material Figure S3) indicate that this indicator is not influenced by a single study. However, the effect size is modest, and the underlying biological mechanisms remain unclear. Therefore, this finding should be interpreted as a potential association rather than a confirmed risk factor.

### Diabetes

**Fig. 5:**
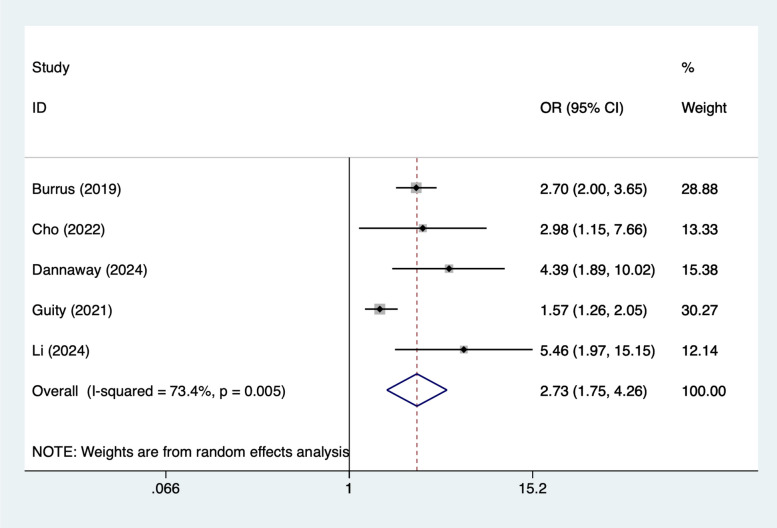
Forest plot of the association between diabetes mellitus and postoperative shoulder stiffness (PSS) after rotator cuff repair. Each square represents the odds ratio (OR) from an individual study, with square size proportional to study weight. Horizontal lines indicate 95% confidence intervals (CIs). The diamond represents the pooled OR and its 95% CI. An OR > 1 suggests that diabetes mellitus was associated with higher odds of PSS

Five articles mentioned diabetes. A heterogeneity test (I^2^ = 73.4%, *P* = 0.005). A random-effects model was used for analysis. The results (Fig. 5) suggest that diabetes was associated with PSS after rotator cuff repair [OR = 2.73, 95% CI (1.75, 4.26)]. A sensitivity analysis was conducted by sequentially excluding studies. The results (Supplementary Material Figure S4) indicate that this indicator is not influenced by a single study.

### Publication bias

This study used funnel plots and the Egger test to assess publication bias. Visual inspection of the funnel plot (Supplementary Materials Figures S5–S8) suggested asymmetry, and Egger’s test indicated potential publication bias for the prevalence analysis (*P* = 0.001). A trim-and-fill (Supplementary Materials Figure S9) analysis was subsequently performed to explore the possible impact of missing studies. Although the adjusted pooled estimate was not materially different from the original estimate, these findings should be interpreted cautiously because trim-and-fill is an exploratory method and cannot fully account for publication bias. The remaining funnel plots were symmetrical for female sex (*P* = 0.19), age < 50 (*P* = 0.068), and diabetes (*P* = 0.724).

### Meta regression

Due to the high heterogeneity of this study, meta-regression was used to explore the specific sources of heterogeneity. Meta-regression analysis (see Supplementary Material Table S2) was conducted to assess whether year of publication, country, sample size, surgical technique, tear characteristics, rehabilitation protocol, timing of stiffness assessment, and the definition of postoperative shoulder stiffness contributed to between-study heterogeneity. Most variables did not show statistically significant associations in the meta-regression model. Country showed a borderline association (*P* = 0.050), whereas the remaining variables were not statistically significant. Nevertheless, because the analysis was based on only eight studies, it was likely underpowered; therefore, these findings should not be interpreted as evidence that these variables are unrelated to heterogeneity.

### Sensitivity analysis

Given the substantially larger sample size of the study by Burrus et al., an additional sensitivity analysis excluding this study was conducted (Supplementary Material Figure S10). After exclusion of the largest study, the pooled prevalence was 19% (95% CI: 11%–27%), with substantial heterogeneity remaining (I^2^ = 96.1%, *P* < 0.001). Compared with the primary analysis, the magnitude of the pooled estimate increased slightly, whereas the overall direction of the findings remained consistent.

## Discussion

This study included eight high-quality cohort studies. The study regions included the Americas, Asia, Europe, and Oceania. The meta-analysis results showed that the overall prevalence of PSS after rotator cuff repair surgery was 17% (95% CI: 9%–24%). Additionally, age < 50 years, female sex, and diabetes were identified as significant risk factors for PSS. Our pooled prevalence of 17% was higher than the 6.4% incidence reported by Baumann et al. [10]. for arthroscopic rotator cuff repair. This difference may be partly explained by differences in the surgical populations included, as our meta-analysis also incorporated studies involving open or mini-open repair. Additional contributors likely include heterogeneity in the definition of postoperative shoulder stiffness and variation in the postoperative time point at which stiffness was assessed. In addition, emerging evidence suggests that the relationship between postoperative stiffness and tendon healing may be complex, although current data remain limited and should be interpreted cautiously. From a clinical perspective, postoperative shoulder stiffness is an important condition that may require additional management and rehabilitation. Therefore, the present findings not only provide an updated estimate of prevalence and associated factors but also contribute to a more comprehensive understanding of the clinical significance of this complication.

Recent literature [12] has suggested a possible “stiffness–healing” paradox after arthroscopic rotator cuff repair, in which early postoperative stiffness may be associated with better tendon healing or lower retear rates. However, this hypothesis remains incompletely understood and should not be interpreted as evidence that postoperative stiffness is desirable, as stiffness may still compromise short-term function and rehabilitation. Saade et al. [14]. recently reviewed the management of shoulder stiffness following rotator cuff repair and reported that rehabilitation, infiltration, and arthroscopic capsular release may all improve postoperative range of motion, although the available evidence remains limited. This management context further supports the clinical importance of identifying patients at increased risk of PSS and highlights the relevance of our study for postoperative follow-up and treatment planning. Recent literature [29] suggests that most postoperative shoulder stiffness cases tend to resolve within 6 months after rotator cuff repair. This natural history may partly explain why prevalence estimates vary according to the timing of assessment. However, early stiffness remains clinically important because it can still affect short-term recovery and rehabilitation.

Regarding the prevalence of PSS, the results showed that the overall prevalence after rotator cuff repair surgery was 17% (95% CI 9%−24%), but there was extremely high heterogeneity (I^2^ = 98.2%), the high degree of heterogeneity observed in the pooled prevalence (I^2^ = 98.2%) highlights the variability among the included studies in terms of patient populations, surgical techniques, definitions of postoperative shoulder stiffness, rehabilitation protocols, and timing of outcome assessment. While the pooled estimate provides a summary measure across studies, its clinical applicability is limited, and the estimate should not be overgeneralized to all patient populations. Sensitivity and subgroup analyses suggest that some of the heterogeneity may be explained by geographic region, publication year, and mean age, but substantial unexplained variability remains. Therefore, the findings should be interpreted primarily as an overall descriptive estimate rather than a precise measure for clinical decision-making. We further conducted a subgroup analysis and found significant differences in prevalence rates across different continental regions: 23% in Asia, 10% in the Americas, 16% in Oceania, and in Europe at 5%. This geographical distribution may be influenced by various factors, such as racial differences, variations in surgical techniques, differences in postoperative rehabilitation methods, and patients’ health awareness and compliance [30]. The higher prevalence in Asia may be associated with factors such as inadequate postoperative management, restricted early activity, or poor patient compliance [31, 32]. In contrast, the more mature postoperative rehabilitation systems and standardized medical pathways in European countries may contribute to lower PSS incidence rates [33]. However, this conclusion should be interpreted with caution, as the sample size for regional differences analysis was relatively limited, and specific differences in surgical techniques, surgeon experience, and rehabilitation mechanisms across regions were not considered [34]. The pooled prevalence of PSS should be interpreted cautiously because the between-study heterogeneity was extremely high. To better explore this issue, we conducted additional subgroup analyses by sample size, publication year, and mean age. Notably, heterogeneity decreased in the subgroup of studies with a sample size < 300, whereas it remained very high in the subgroup with a sample size ≥ 300, suggesting that variation in study scale may have contributed to the overall heterogeneity. In contrast, subgrouping by publication year and mean age did not materially reduce heterogeneity. This pattern suggests that the observed variability is unlikely to be explained by a single factor alone and may instead reflect differences in diagnostic criteria, follow-up time points, operative techniques, rehabilitation strategies, and baseline patient characteristics across the included studies. Accordingly, the pooled prevalence should be regarded as an exploratory summary of the current evidence rather than a precise epidemiological estimate.

Although subgroup analysis suggested variation in prevalence across geographic regions, these findings should be interpreted with extreme caution. Some regional subgroups were based on only one or two studies, which substantially limits the reliability and statistical robustness of such comparisons. Therefore, the observed differences are more likely to reflect variations in study design, patient populations, diagnostic criteria, surgical techniques, rehabilitation protocols, and timing of outcome assessment, rather than true geographic effects.

In terms of age-related factors, In the pooled analysis, age < 50 years was associated with a slightly increased likelihood of PSS after rotator cuff repair. However, the magnitude of this association was modest, and the heterogeneity across studies was substantial [35]. Therefore, this finding should be interpreted cautiously. Although younger age has been reported in some previous studies as a possible associated factor, the currently available evidence is insufficient to support strong mechanistic explanations. Accordingly, younger age is better regarded as a potential associated factor rather than a definitive or clinically decisive predictor of PSS [36]. Female sex was associated with a modest increase in the likelihood of postoperative shoulder stiffness (OR = 1.66). Although this association reached statistical significance, the effect size is small, and its clinical relevance is likely limited [37]. The underlying biological or mechanistic basis for this association remains unclear, as the included studies did not provide sufficient data to explore hormonal, anatomical, or other contributing factors. Consequently, this finding should be interpreted cautiously as an observational association rather than a definitive risk factor [38].

Diabetes, as a systemic metabolic disease, has been identified by multiple studies as a high-risk factor for poor postoperative recovery [39]. This study showed that the risk of PSS was significantly increased in diabetic patients (OR = 2.73), suggesting that preoperative assessment and management of such patients should be strengthened. Diabetes affects the tissue healing process through multiple mechanisms, including enhanced inflammatory responses, increased collagen deposition, inhibited angiogenesis, and accelerated fibrosis under hyperglycemic conditions, all of which may exacerbate postoperative shoulder joint stiffness [40, 41]. Although diabetes mellitus was identified as a strong risk factor for postoperative shoulder stiffness, the underlying mechanisms and the influence of clinical variables such as glycemic control, disease duration, and treatment status remain unclear. The included studies did not consistently report these variables, limiting the depth of our analysis. Consequently, the observed association should be interpreted as an overall observational association rather than a definitive causal effect.

The exploratory meta-regression did not identify clear and robust study-level determinants of heterogeneity, although country showed a borderline association. However, this finding should be interpreted cautiously. With only eight included studies, the meta-regression was likely underpowered, and the absence of statistically significant associations for most variables should not be interpreted as evidence that these factors are not important sources of heterogeneity. Rather, clinically relevant differences in surgical technique, tear characteristics, rehabilitation protocol, timing of assessment, and the definition of postoperative shoulder stiffness may still have contributed to the substantial between-study variability. This suggests that the factors contributing to study heterogeneity may be more complex and may be related to surgical technique selection, surgical approach, surgeon skill level, postoperative rehabilitation protocols, and patient-specific factors (such as comorbidities, occupation, and physical activity level), which are rarely uniformly reported or controlled for in existing studies. Additionally, some studies did not strictly define stiffness (e.g., criteria for restricted range of motion, subjective evaluation of stiffness), which may have exacerbated inconsistencies in the results. In addition to the substantial heterogeneity observed, evidence of publication bias was also detected in this study. Funnel plot asymmetry and Egger’s test suggested that smaller studies with less extreme or non-significant findings may be underrepresented. Although trim-and-fill analysis was performed to explore the potential impact of missing studies, this approach has methodological limitations and cannot fully correct for bias. Therefore, both heterogeneity and potential publication bias should be considered when interpreting the pooled prevalence and associated factors, and the results should be viewed as approximate rather than definitive estimates.

From a clinical perspective, the present findings may help raise awareness of postoperative shoulder stiffness as a relatively common complication after rotator cuff repair. However, given the substantial heterogeneity, variability in diagnostic definitions, and the observational nature of the included studies, these results should not be interpreted as a basis for definitive clinical recommendations. Instead, they may be useful for identifying areas where closer monitoring and further research are warranted.

### Strengths and limitations

The strengths of this study are as follows: first, it included eight high-quality cohort studies with a total sample size exceeding 20,000 participants, covering multiple countries and regions, ensuring good representativeness and broad external validity; second, it employed rigorous systematic review and meta-analysis methods, combined with the NOS score to assess literature quality, ensuring data reliability; Third, sensitivity analysis, subgroup analysis, and meta-regression were used to explore the sources of heterogeneity, while funnel plots and Egger’s test were employed to assess publication bias, thereby enhancing the robustness and credibility of the results.

However, this study also has certain limitations. First, all included studies were observational, lacking randomized controlled trials, making it difficult to completely rule out the influence of confounding biases; Second, the definition criteria for PSS vary across studies and rely partially on subjective evaluations, which may affect the consistency of results; Third, important influencing factors such as surgeon experience, surgical technique differences, rehabilitation protocols, and compliance were not detailed in the original studies, limiting the in-depth exploration of the mechanisms underlying PSS; Finally, despite subgroup and regression analyses, significant heterogeneity persists between studies, suggesting the presence of unidentified potential confounding factors. All included studies were observational in nature, which inherently introduces a risk of confounding. Although adjusted effect estimates were used whenever available, the covariates included in the multivariable models differed across studies. As a result, residual confounding cannot be excluded, and the associations observed in this meta-analysis should be interpreted as observational rather than causal.

### Future research directions

Future research should focus on conducting multicenter, randomized controlled trials to further validate the risk factors and intervention measures for shoulder stiffness following rotator cuff repair surgery, standardize the definition and assessment criteria of PSS, explore its underlying mechanisms, and design individualized rehabilitation programs tailored to high-risk populations (individuals under 50 years of age, women, and diabetic patients). Additionally, studies should compare the effects of different surgical techniques and rehabilitation strategies, conduct long-term follow-up assessments to evaluate the sustained impact of PSS on shoulder joint function, and investigate the roles of genetic and molecular markers to provide scientific evidence for precision treatment and prevention.

## Conclusion

The available evidence suggests that postoperative shoulder stiffness after rotator cuff repair is not uncommon. Although variation in reported prevalence was observed across studies and regions, these findings should be interpreted cautiously because of substantial heterogeneity and limited data in some subgroups. Age < 50 years, female sex, and diabetes were identified as factors associated with postoperative shoulder stiffness, although the effect size for age < 50 years was small and the observational nature of the evidence should be considered. These findings may help inform clinical awareness, but further well-designed prospective studies are needed before firm conclusions or specific clinical recommendations can be made.

## Supplementary Information

Supplementary Material 1.

## Data Availability

All the data are included herein (main text and supplementary section).

## Abbreviations

PSS: Postoperative shoulder stiffness
NOS: Newcastle-Ottawa Scale
OR: Odds ratios
CI: Confidence intervals
