# Supplementary material for: Prevalence and risk factors of shoulder stiffness after rotator cuff repair: a meta-analysis
Source: BMC Musculoskelet Disord. 2026 May 5;27:543. doi: 10.1186/s12891-026-09856-0 (PMC13312692; doi:10.1186/s12891-026-09856-0)
Supplement: Supplementary file 1 — Supplementary Material 1. [file 12891_2026_9856_MOESM1_ESM.docx]

Table S1 specific search strategy

((("Rotator Cuff Injuries"[Mesh]) OR (((((((((((((((((((((Rotator Cuff Injuries[Title/Abstract]) OR (Cuff Injury, Rotator[Title/Abstract])) OR (Injury, Rotator Cuff[Title/Abstract])) OR (Rotator Cuff Injury[Title/Abstract])) OR (Rotator Cuff Tears[Title/Abstract])) OR (Rotator Cuff Tear[Title/Abstract])) OR (Tear, Rotator Cuff[Title/Abstract])) OR (Tears, Rotator Cuff[Title/Abstract])) OR (Rotator Cuff Tendinitis[Title/Abstract])) OR (Rotator Cuff Tendinitides[Title/Abstract])) OR (Tendinitis, Rotator Cuff[Title/Abstract])) OR (Rotator Cuff Tendinosis[Title/Abstract])) OR (Rotator Cuff Tendinoses[Title/Abstract])) OR (Tendinoses, Rotator Cuff[Title/Abstract])) OR (Tendinosis, Rotator Cuff[Title/Abstract])) OR (Glenoid Labral Tears[Title/Abstract])) OR (Glenoid Labral Tear[Title/Abstract])) OR (Labral Tear, Glenoid[Title/Abstract])) OR (Labral Tears, Glenoid[Title/Abstract])) OR (Tear, Glenoid Labral[Title/Abstract])) OR (Rotator Cuff Repair[Title/Abstract]))) AND (("Vascular Stiffness"[Mesh]) OR (Stiffness[Title/Abstract]))) AND (("Risk Factors"[Mesh]) OR (((((((((((((((((((Risk Factors[Title/Abstract]) OR (Factor, Risk[Title/Abstract])) OR (Risk Factor[Title/Abstract])) OR (Population at Risk[Title/Abstract])) OR (Populations at Risk[Title/Abstract])) OR (Risk Scores[Title/Abstract])) OR (Risk Score[Title/Abstract])) OR (Score, Risk[Title/Abstract])) OR (Risk Factor Scores[Title/Abstract])) OR (Risk Factor Score[Title/Abstract])) OR (Score, Risk Factor[Title/Abstract])) OR (Health Correlates[Title/Abstract])) OR (Correlates, Health[Title/Abstract])) OR (Social Risk Factors[Title/Abstract])) OR (Factor, Social Risk[Title/Abstract])) OR (Factors, Social Risk[Title/Abstract])) OR (Risk Factor, Social[Title/Abstract])) OR (Risk Factors, Social[Title/Abstract])) OR (Social Risk Factor[Title/Abstract])))

Table S2 Meta regression

| Variable | Coef. | Std. Err. | P | 95%CI |
| --- | --- | --- | --- | --- |
| Year of publication | 0.005 | 0.012 | 0.718 | (-0.025, 0.034) |
| Country | 0.224 | 0.091 | 0.050 | (0.001,0.448) |
| Sample size | -0.005 | 0.546 | 0.131 | (-0.001, 0.037) |
| Surgical technique | 0.24 | 0.133 | 0.785 | (-0.651, 0.065) |
| Tear characteristics reported | 0.161 | 0.352 | 0.273 | (-0.552, 0;712) |
| Rehabilitation protocol | 0.961 | 0.186 | 0.119 | (-0.131, 0.176) |
| Timing of stiffness assessment | 0.239 | 0.881 | 0.293 | (-0.132, 0.111) |
| Definition of postoperative shoulder stiffness | 0.752 | 0.752 | 0.174 | (-0.132, 0.237) |


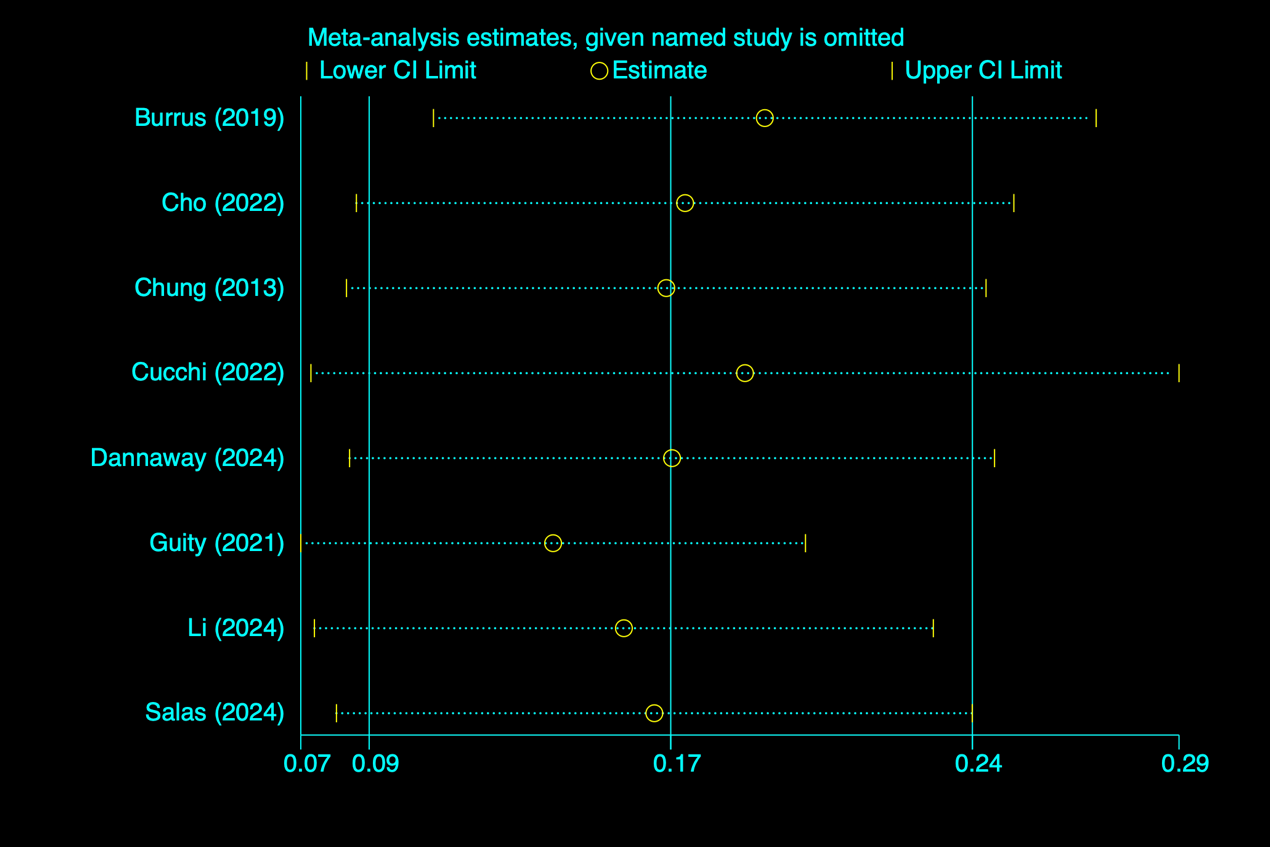


Figure S1 Sensitivity analysis of prevalence rate


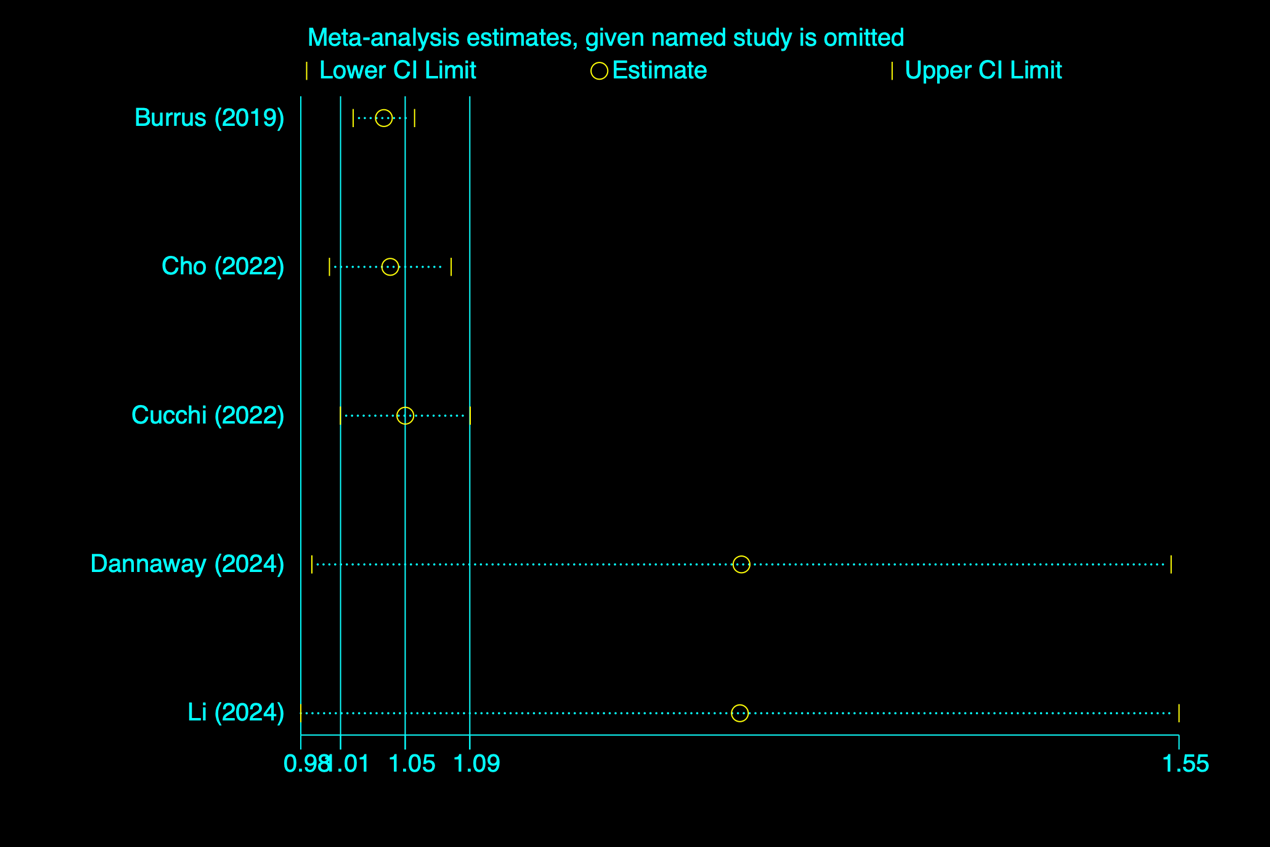


Figure S2 Sensitivity analysis of age<50


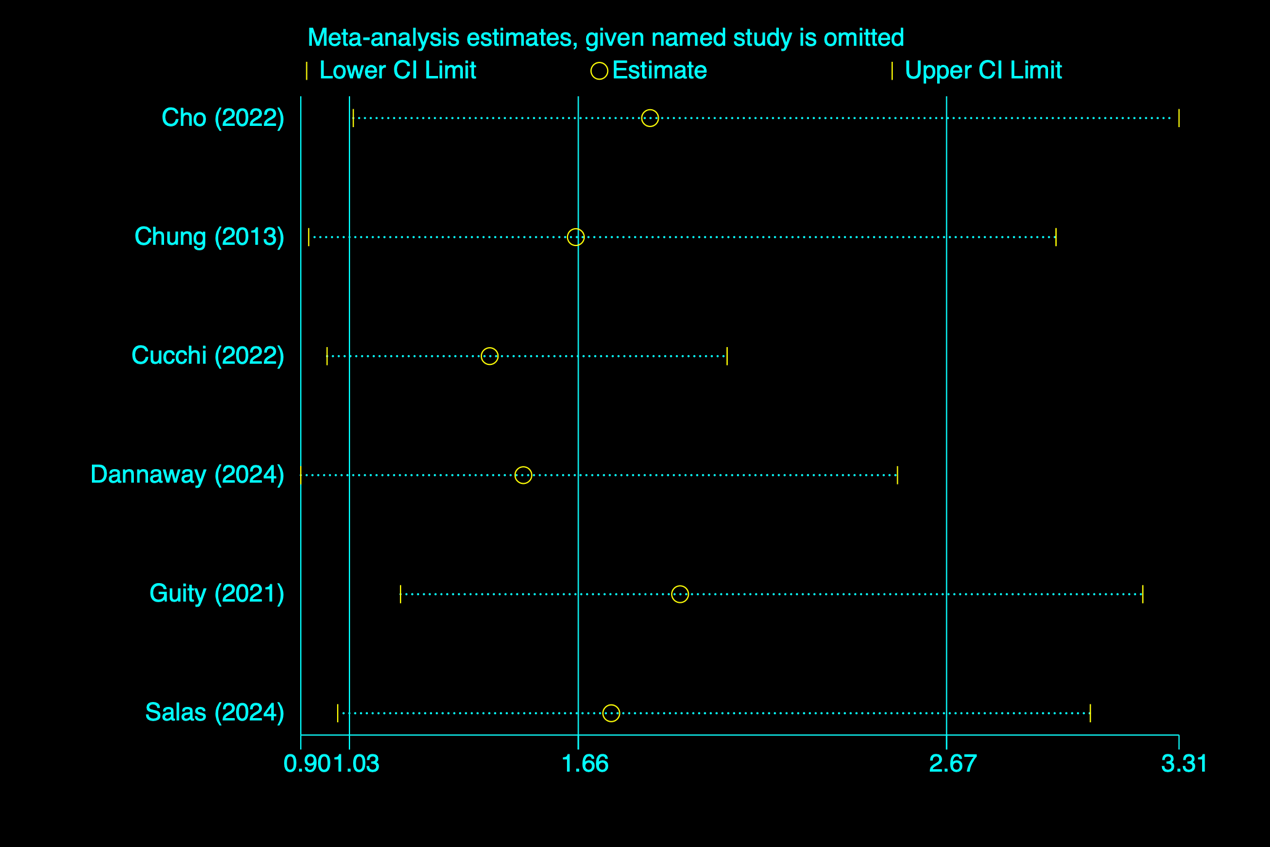


Figure S3 Sensitivity analysis of female


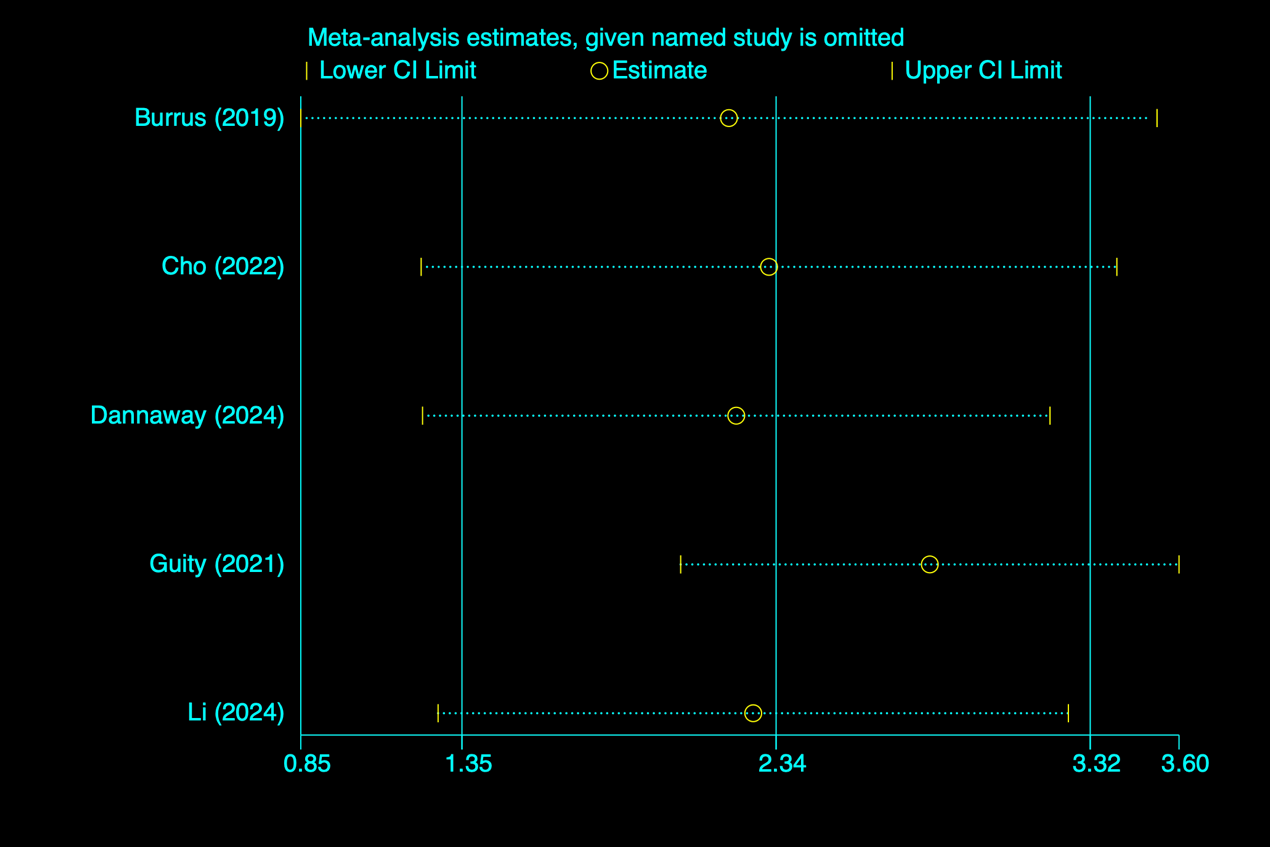


Figure S4 Sensitivity analysis of diabetes


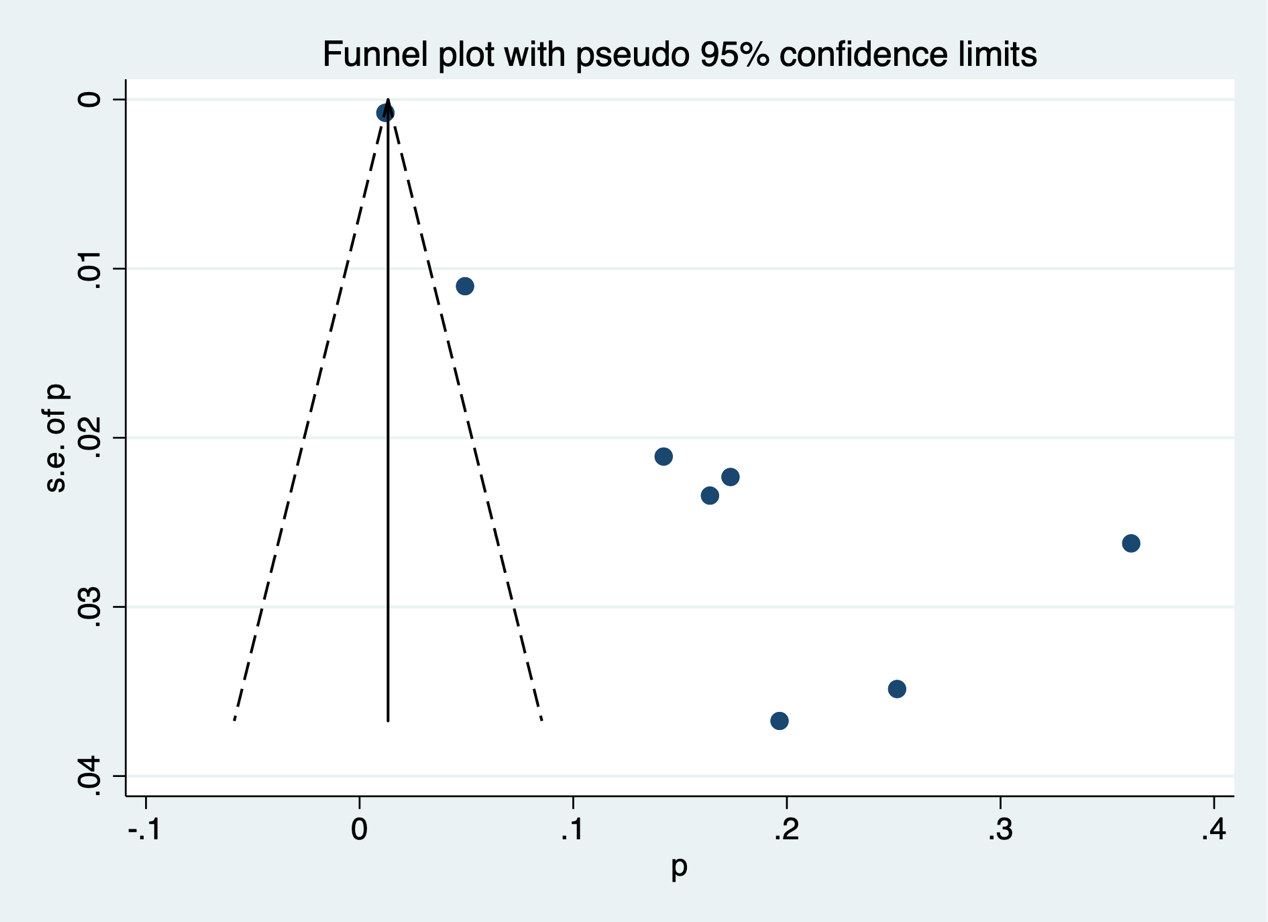


Figure s5 Funnel plot of prevalence rate


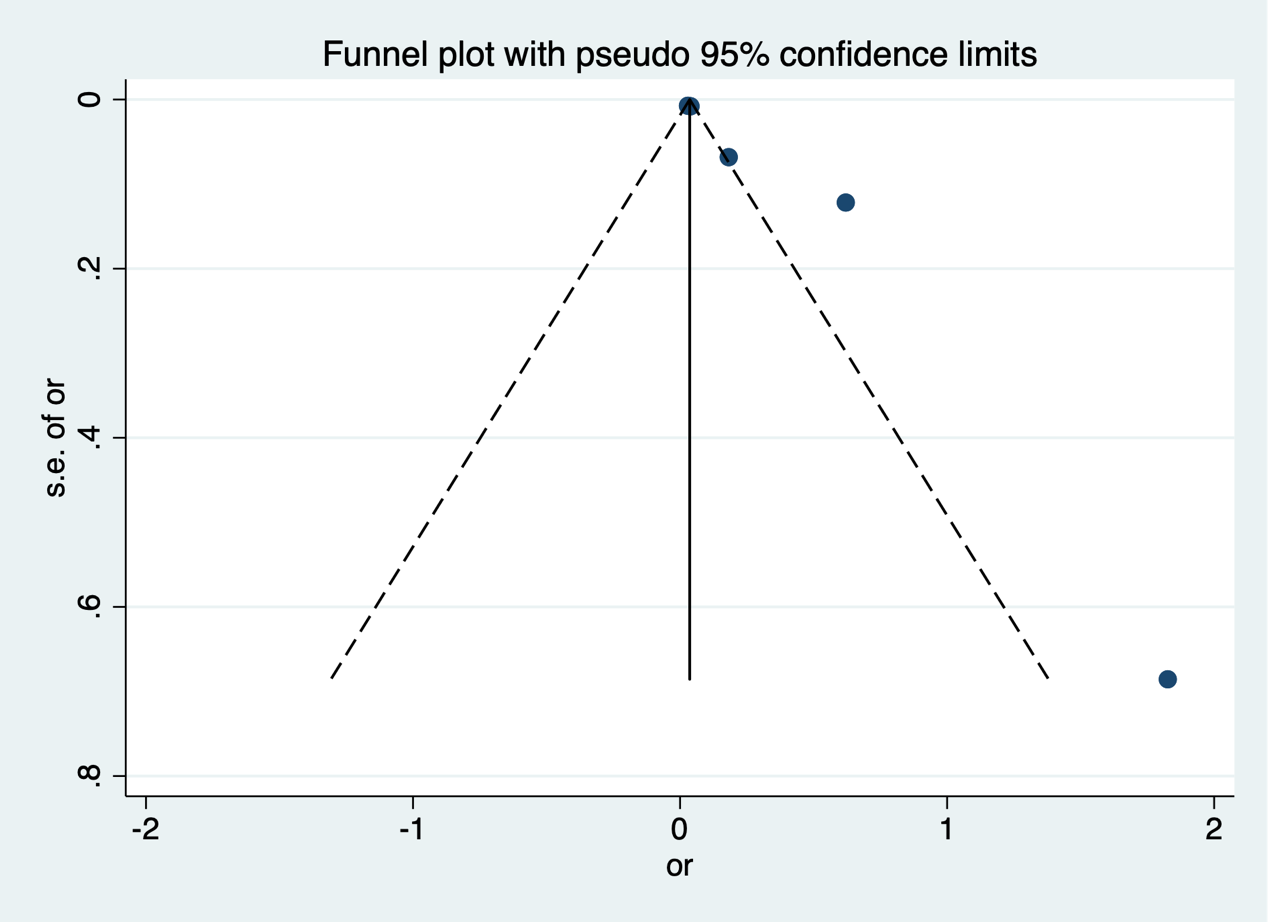


Figure s6 Funnel plot of age<50


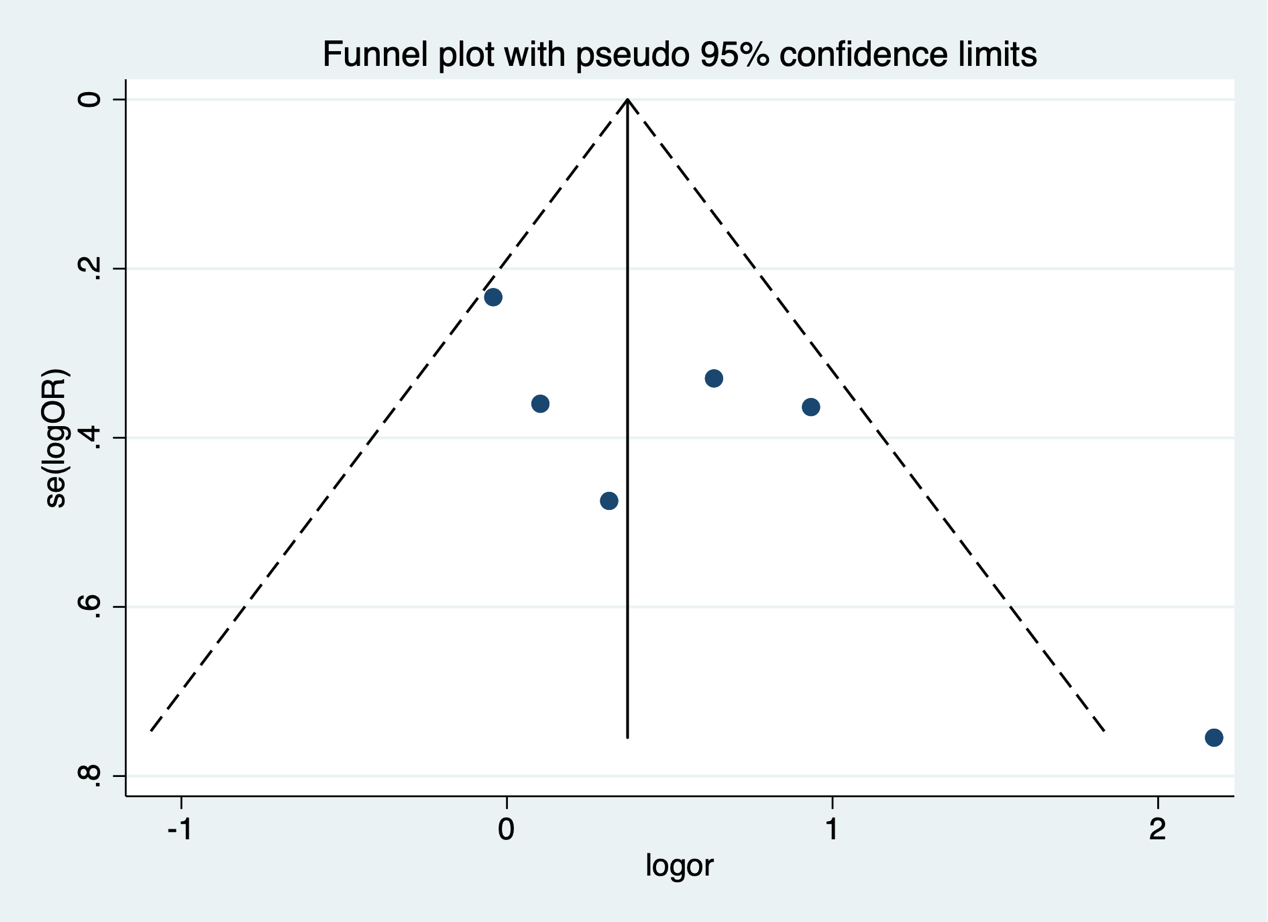


Figure s7 Funnel plot of female


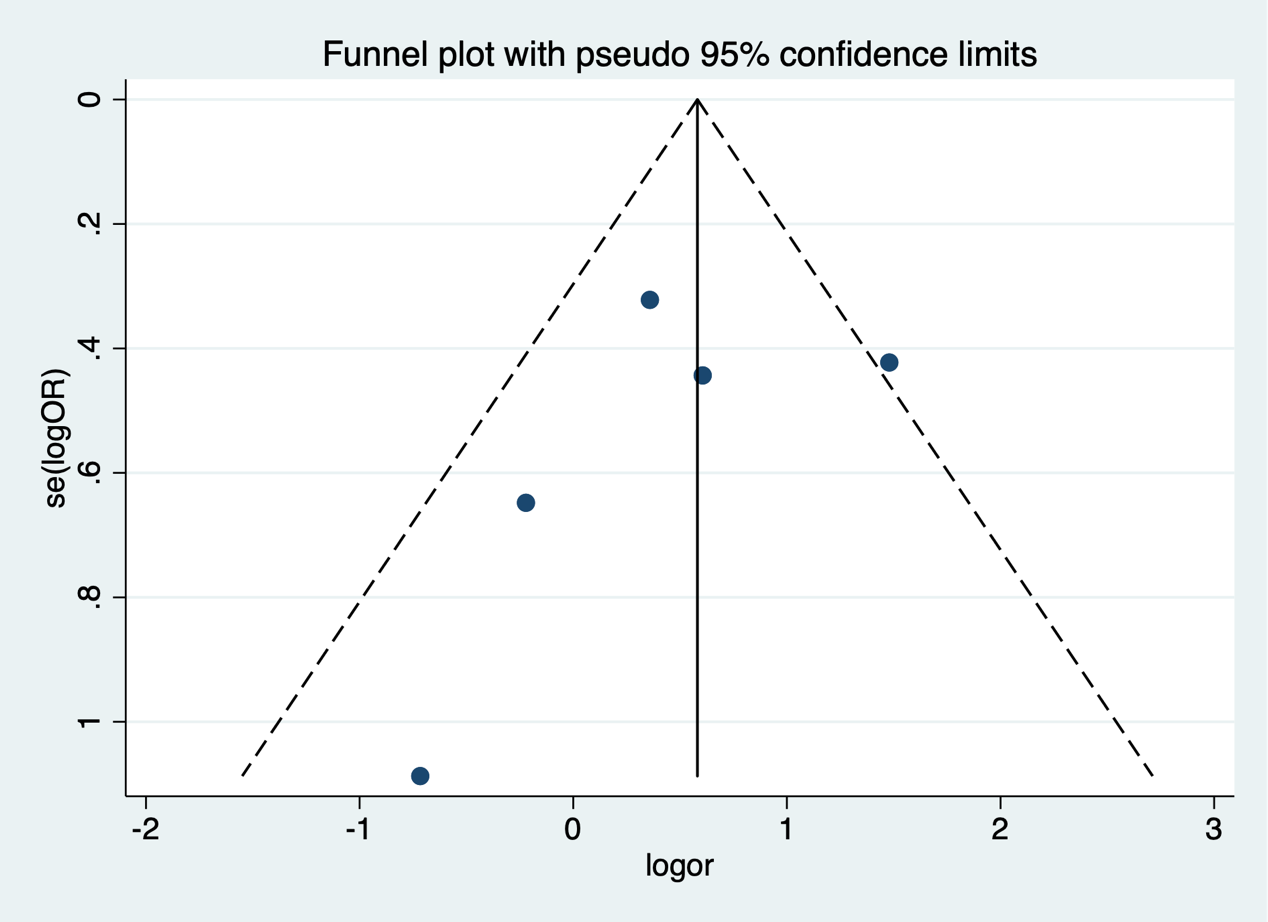


Figure s8 Funnel plot of diabetes


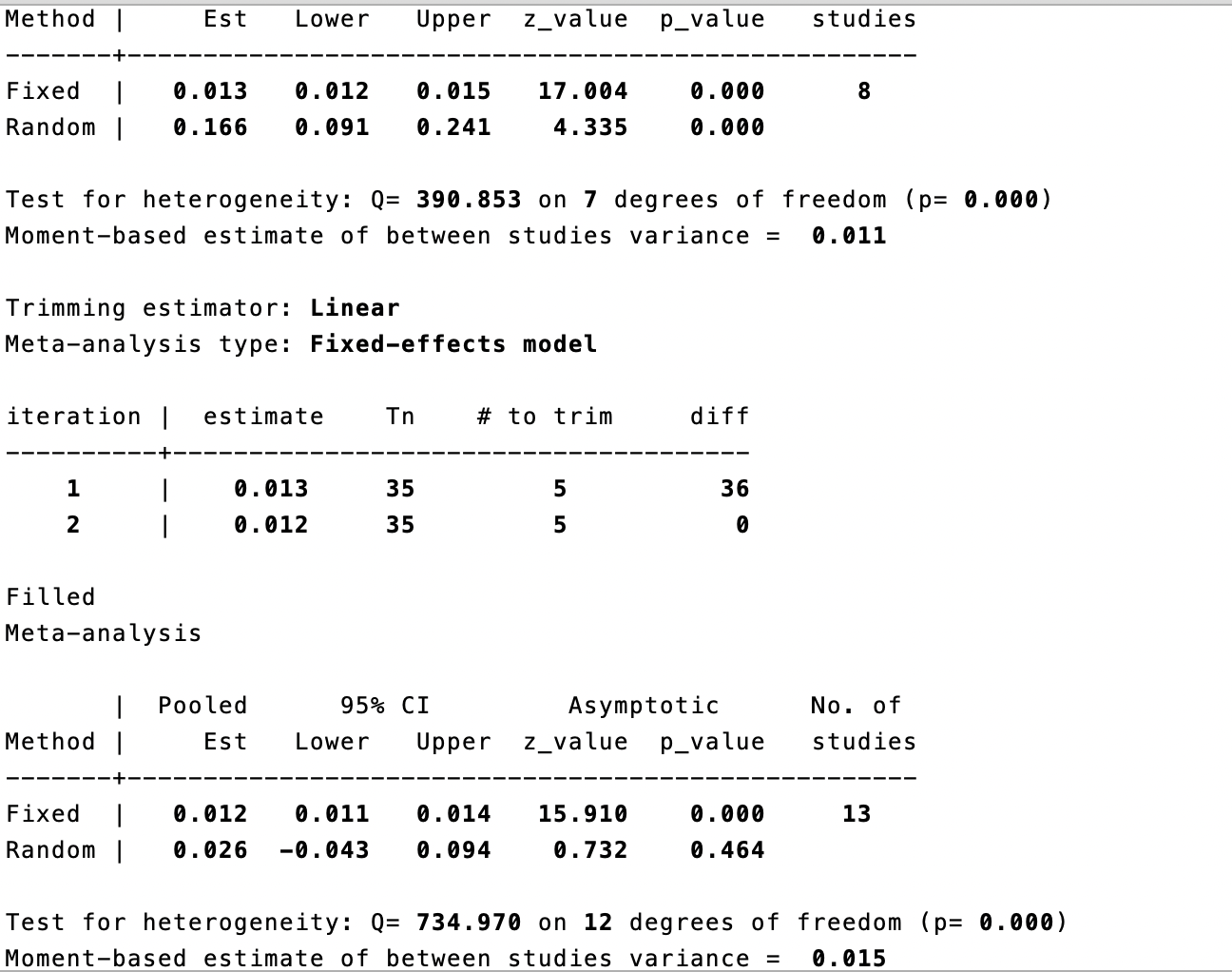


Figure s9 trim-and-fill results of prevalence rate


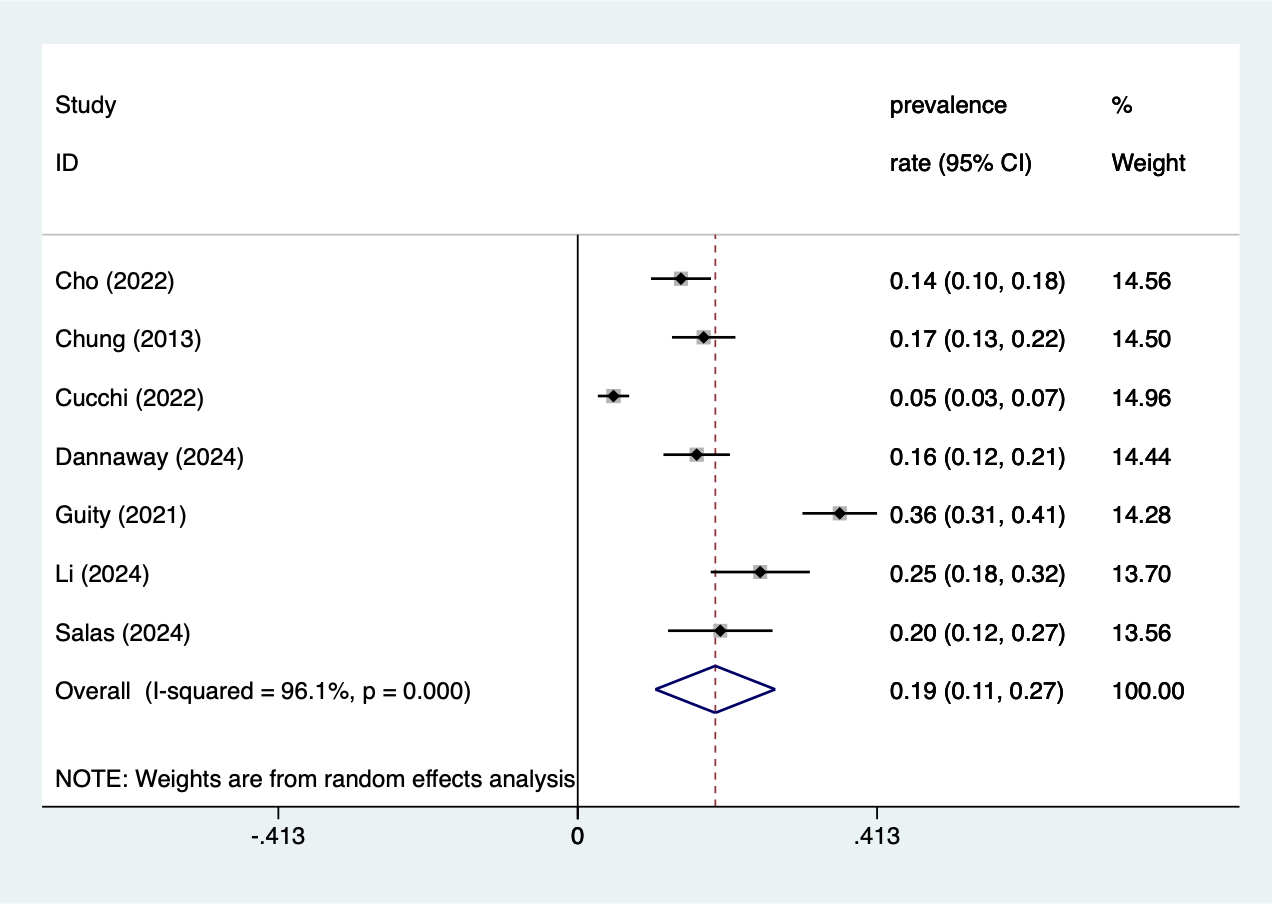


Figure S10 an additional sensitivity analysis excluding Burrus et al.,2019
